# Surgical and Pathological Cases

**Published:** 1832-02

**Authors:** James Laidlaw

**Affiliations:** Member of the Royal Colleges of Surgeons of London and Edinburgh, and formerly House Surgeon to the Middlesex Hospital.


THE LONDON

### Medical and Physical Journal.

396, VOL. LXVII.] FEBRUARY 1832. [68, *New Series*.

"For many fortunate discoveries in medicine, and for the detection of numerous errors, the world is indebted to the rapid circulation of Monthly Journals; and there never existed any work to which the Faculty, in Europe and America, were under deeper obligations than to the 'Medical and Physical Journal of London,' now forming a long but invaluable series."—RUSH.

### ORIGINAL PAPERS, AND CASES

### OBTAINED FROM PUBLIC INSTITUTIONS AND OTHER AUTHENTIC SOURCES.

### SURGICAL AND PATHOLOGICAL CASES.

*Surgical and Pathological Cases.* By JAMES LAIDLAW, Esq., Member of the Royal Colleges of Surgeons of London and Edinburgh, and formerly House Surgeon to the Middlesex Hospital.

(Continued from page 14.)

*Wound penetrating the Knee-joint.*

MICHAEL MORAN, fifty years of age, was brought to the hospital, at two o'clock on the morning of the 26th of April, 1829, with a wound three inches in length, extending across the front of the knee-joint, laying the patella bare, and perforating the capsule of the joint on either side of it. At the time of his admission, he was half drunk, complained of great pain in the wound, and continually tossed the limb about in a stupid restless manner, so that it was not without some difficulty a careful examination of it could be obtained. The edges of the wound were found to be much bruised and ragged; there was very little bleeding, but there was a good deal of ecchymosis of the surrounding parts. He said the wound was inflicted by his falling down stairs, and striking his knee against the edge of one of them.

He was placed in bed, and made to lie on his back so as to extend the limb, which was then placed in junks, to prevent its being moved from its position; the wound was neatly brought together by adhesive straps, and leeches applied to the surrounding bruised surface. A dose of jalap and calomel was then given him, and he was warned of the danger which is frequently attendant upon wounds penetrating the great joints.

The following day he complained of severe pain in the kneejoint; but as there appeared to be no swelling whatever of the thigh or leg, and very little around the joint, nothing further was thought necessary than the application of cold lotion to the limb. In a day or two the wound was dressed, and was observed to be granulating healthily; he had no pain, or other symptom of inflammation of the joint, and in the course of a very short time he was dismissed quite well.

In this case every thing was disadvantageous to the patient at the commencement; that is to say, when his age, habits, and actual inebriation at the time of receiving the accident, are considered, to say nothing of the lacerated state of the wound, and the injury done to the surrounding parts, yet he had not a bad symptom, and the wound healed without any violent inflammation, or constitutional irritation. I have introduced the case principally for the sake of contrasting it with the following, in which, notwithstanding the apparently favorable circumstances, very high constitutional irritation ensued to the great jeopardy of the patient.

*Case II. of Wound penetrating the Knee-joint.*

George Phillips, aged forty, was brought to the hospital on the 19th July, 1829, in consequence of a severe wound he had received in one of his knees. It appeared he was working at his trade as a cooper, and in using the spokestrave, he was steadying the timber he was at work upon, by pressing against it with his knee, when the instrument slipped, and came, with all the force he was applying to the work, in contact with the knee, and inflicted the wound.

Upon examining the limb, I found a wound extending across the patella, two inches in length, and perforating the capsule of the joint on its inner side: the wound in the capsular ligament was about half an inch in length, and had permitted a quantity of blood to flow into the joint, where it had coagulated. This was carefully removed, and the edges of the wound were brought together by adhesive straps; and, as it was a clean incised wound, they approximated very neatly; the leg was rolled, and a slender splint placed behind the knee, so as to keep the limb extended; and still more to guard against any accidental motion, it was placed in junks: the nurse was directed to keep the bandages wet with cold lotion.

He thus continued very comfortable and free from pain, till the following morning, when he said he felt very uneasy, and was anxious to have the bandages undone and the wound examined. In order to gratify him, as he appeared very nervous and whimsical, this was complied with, and the inner end of the cut, over the wound in the joint, appeared to be already united; the surrounding parts felt very hot, and there was a slight degree of swelling about four inches above the knee, on the inside of the thigh, and which he said gave him great pain when touched: there was no swelling of the joint. Leeches were applied to the swelling in the thigh, and a dose of calomel and colocynth was administered.

These means allayed the irritation only for the moment: on the following day every symptom became more alarming; the swelling had increased so as to occupy nearly the whole of the thigh; the integuments were covered with an inflammatory redness, and were very hot; the pain was severe, and a considerable degree of fever had already come on; his tongue was white, and his pulse full and frequent; he complained of the heat and thirst as intolerable. It was ordered that twenty-five leeches should be applied to the thigh, and that it should be kept constantly wet with cold lotion. A saline draught, with twenty minims of the Liq. Antim. Tart., was ordered to be given every four hours, and a pill of Calomel and Pulv. Antim. at night.

The following day he was much better: the swelling of the limb had in a great measure subsided, and there was no longer that great heat and thirst which had been so distressing; the pulse had fallen considerably, and the skin in general felt moist and healthy. He was directed to continue the medicine.

July 28th. Every thing continued going on well: the wound was poulticed, and suppurated healthily, the matter evidently coming from beneath the integuments only, and not from the joint. The inflammatory blush and swelling of the limb still continue, and there is occasionally a considerable degree of heat. His general health is much disordered; his tongue white and furred, and the pulse small and frequent. He was ordered to have a pill of calomel and rhubarb occasionally at night, and to omit the antimony in the draught.

July 30th. Upon carefully examining the limb, a quantity of matter was discovered, burrowing beneath the integuments of the thigh, and which could be pressed out at the wound in the knee. A probe was passed from the wound, and the cavity of the abscess was found to be very extensive beneath the fascia of the thigh: about seven ounces of matter were pressed out. It was thought better to make an opening in the superior part of this abscess, to admit of the more ready escape of the pus, and to prevent its burrowing further amongst the muscles: and an incision was accordingly made in it with an abscess lancet, through which the matter flowed readily. He now suffers very little pain; his general health is better, and he has a good appetite.

He continued in this condition, with very little variation, during the whole of the month of August, the matter discharged from the abscess being sometimes in greater and at other times in smaller quantity. Tonic medicines and a liberal diet were given him, and he had a small allowance of wine and porter; his appetite continued good, and, with the exception of an occasional feeling of great debility, his general health seemed to be unimpaired. At one period the leg was attacked with violent inflammation, and an abscess seemed to have formed immediately below the head of the tibia, between the integuments and the tendons of the sartorius and gracilis muscles. This subsided without being opened, or giving rise to any unpleasant symptoms. Toward the latter end of the month the discharge became less in quantity; and by the 3d of September, it had entirely ceased. The limb had then a hard, knotty, irregular feel along the course of the abscess, but was quite free from pain. There was much œdema of the leg and foot, and the knee-joint could not be bent. Being thus in hopes that he would speedily become convalescent, it was observed with considerable regret, on the 8th of September, that a fresh attack of inflammation had taken place without any apparent cause, and, notwithstanding the adoption of very active antiphlogistic measures, suppuration again took place, and the discharge became as profuse as it had been six weeks before.

The poor fellow, being now greatly debilitated by long confinement, became fretful and discontented; his hopes of ultimate recovery deserted him, and conceiving it would be more comfortable to die at home, surrounded by his friends, he insisted upon being removed, and he was taken away from the hospital shortly afterwards. How the case finally terminated I have been unable to learn.

Taking the above two cases together, I think they afford a fair example of the great uncertainty of our prognosis, in wounds penetrating the cavity of the joints. In as far as the wound of the particular joint was concerned, these cases were precisely similar, but the circumstances of the patients were widely different. In the first case, a drunken, dissipated fellow, advanced in life, and while under the influence of liquor, received a severe lacerated wound, attended with much injury to the surrounding parts. Who could have expected such a case would do well? yet so it turned out; while poor Phillips, who was a steady, industrious, healthy man, in the prime of life, receives a wound in a similar situation from a clean, cutting instrument, and immediately obtains surgical assistance; yet, with every attention, after months of confinement, barely escapes (if he even did so ultimately) with his life.

*Rupture of the Kidney.*

October 11th, 1829. Alexander Elliot, a remarkably fine healthy young man, twenty-one years of age, while running at full speed, came unexpectedly in contact with the iron palisades of one of the squares, and struck his abdomen against them with such force that he was thrown to the ground by the shock; he immediately got up, and not feeling himself, at the moment, much hurt, walked home, a distance not greater than a hundred yards. Soon after he got home, he became very sick, and vomited, but did not consider it of much consequence, till about two hours afterwards, when, having occasion to make water, he found nothing but blood flowed from the bladder; and he then became alarmed, and, procuring a coach, had himself conveyed to the hospital.

At the time of his admission, when I first saw him, he was pale, cold, and covered with a clammy perspiration; his countenance was anxious, and he was greatly agitated. Upon a superficial view, he appeared far more alarmed than injured. He complained of pain in the belly, particularly in the right hypochondriac region; his pulse was full and regular, at seventy-five. At his request some milk and water was given to him; but, immediately upon drinking it, he vomited it up. Twenty leeches were applied to the abdomen without delay.

In the evening the symptoms became more alarming; the vomiting increased, and was accompanied with constant hiccup, which greatly distressed him; the pain in the belly was also much greater; he rolled about in the bed, was very irritable, and restless; the anxiety of countenance was much increased, and the respirations were more frequent than natural: he did not complain when the abdomen was pressed with the hand. As he had not made water since his admission, I introduced a catheter, and drew off about ten ounces of fluid, apparently chiefly blood, and directed that fifteen more leeches should be applied to the belly.

Upon visiting him early the following morning, (Oct. 12th,) he said he was somewhat easier, but had passed a miserable, restless night, and had been greatly distressed by the constant vomiting; his countenance was still very anxious, and he appeared to be suffering a good deal from alarm and agitation. A catheter was introduced, and twelve ounces of bloody urine drawn off: this he said greatly relieved him. Towards night he became much worse; the belly was swollen and tense, and he complained of great pain upon the application of the hand; the vomiting was not abated, and it distressed him much more in consequence of the tendernesss of the abdomen. It was found necessary to continue the use of the catheter, and the urine was deeply tinged with blood. Pulse small, sharp, and regular, its frequency not increased; tongue natural; skin hot and dry; and he complained of great thirst, which could be only partially allayed by moistening his mouth, as every thing he swallowed was instantly rejected. ℞. Hydrarg. Submur. gr. ij.; Pulv. Opii, gr. ss. statim. Haust. Potassæ Citrat. quartâ quâque hora.

October 13th. Somewhat better; he has had some sleep during the night, and is greatly refreshed; the belly is less painful, and the swelling is diminished: he has passed about a pint of urine without the catheter. As his bowels were confined, he was ordered an enema, and it has produced a copious evacuation, which he says has made him feel much more comfortable. Pulse small, sharp, and much increased in frequency; tongue white and moist. V.S. ad ℥xij. To continue the medicines.

October 14th. He is greatly improved; his skin is cooler, and there is less irritability in the pulse; his bowels have been opened several times, and he passes his water naturally; he suffers very little. The blood drawn yesterday cupped and buffed. Ordered V.S. ad ℥x. Haust. Acetat. Ammon. c̄ Liq. Antim. Tart. ♏x. quartâ quâque hora.

October 16th. Since the day before yesterday he has been gradually getting better: the pain in the belly is almost gone, and the only thing he now complains of is great thirst and an uncomfortable fulness of the belly. The blood which was taken on the 14th exhibited no inflammatory appearance.

For several days after the last report, every thing appeared to be going on favorably, and he seemed to be getting well rapidly, when suddenly, on the evening of the 21st October, he became much worse; his skin again became hot and dry; his tongue parched and covered with a brown fur; and the anxiety of countenance, which was so apparent at first, had again appeared; he complained of great pain in the right lumbar region; the pulse was full and frequent, but regular. As his bowels had not been opened during the day, an enema was ordered, and he was directed to take the following powder every four hours: ℞. Pulv: Rhei, gr. v.; Hydrarg. c̄ Cretâ, gr. iij. M. fiat pulv.

On visiting him the following morning, (October 22d,) I found him in less pain than the preceding evening, but the other unfavorable symptoms were unabated. In the course of the day he had a severe shivering fit, which lasted for several minutes, and the pulse became very small and quick. Upon examining the belly, the part originally injured seemed to incline to the side, and appeared to contain fluid. He was directed to continue the medicines, and to have hot fomentations to the belly.

He continued in nearly the same condition during the two following days, (October 23d and 24th,) appearing only to get a little weaker; but on the morning of the 26th, it was evident that there was no hope of saving him, as he was sinking rapidly. He complained of no pain in any part; he was perfectly sensible and collected, frequently saying that he had made up his mind to die; his countenance was sallow and very anxious, and he appeared to breathe with great difficulty; the pulse was too quick to be counted; the tongue was black and dry, and the skin hot, and without the slightest moisture. He fancied he should like some soda water, and it was accordingly given him, with small quantities of wine occasionally. At seven o'clock in the evening he died, having survived the accident just fourteen days.

The body was examined eighteen hours after death. Upon opening the cavity of the abdomen, the intestines appeared of a dark sooty colour, which was all that was remarkable in them. The whole of the solid viscera were in a healthy condition, with the exception of the right kidney, which, as had been anticipated, was found to be the seat of the mischief: by the injury it had been broken across just below the pelvis, and in it and the surrounding cellular membrane a large abscess was formed, which contained adout three pints of fluid of a light brown colour: this was supposed to be pus mixed with urine.

*Wound of the Throat.*

John Rowe, æt. thirty, by trade a tailor, rose from bed early on the morning of the 5th March, 1829, and, taking a razor from a cupboard in the room, cut his throat: his wife, being in bed in the same room at the time, saw what had happened, and by her cries soon brought the neighbours to her assistance, and they immediately conveyed him to the hospital. I was called up to attend him, and found him in the surgery, quite blanched, and nearly dying from loss of blood: there was an extensive wound in the throat, extending from immediately below the mastoid process of the temporal bone of the left side, to the angle of the jaw on the opposite side. The left sterno-mastoid muscle was laid completely bare; the os hyoides and epiglottis were separated by the incision from the cartilages of the larynx; and the pharynx was so completely cut across, that the vertebræ of the neck were exposed, and could be seen through the gaping wound. There was no bleeding from any particular vessel at the time of his admission, but a general oozing of blood from the wound, which very soon stopped. A ligature was passed through the edges of the incision at both its extremities, and tied, and it was further supported by strapping. No suture could be made in front, as the patient's respiration was greatly impeded upon any attempt to close the wound more completely. The chin was brought to the breast, and there secured by a bandage passed round the head and under the arms, and he was supported in bed by means of a bed-chair.

He very soon rallied from the apparently dying condition in which I first saw him, and became calm and composed, making many attempts to speak, as if desirous of communicating something to us which he considered of importance: these attempts were, of course, unsuccessful. The day after his admission, he signified by signs that he could write on a board any thing he wished to ask us; and, upon being furnished with chalk, he inquired, with great apparent earnestness, if we did not think there was some hope of his ultimate recovery. He also complained of great thirst, which he endeavoured to allay by endeavouring to drink water, but which merely passed from the mouth out again at the wound: he said it afforded him very little relief. I explained to him the necessity of introducing a tube for the purpose of conveying food to the stomach, and he expressed his willingness to submit to it. On the third day an attempt was made to pass the gum elastic œsophagous tube through the mouth into the gullet, but, after several trials, it was found to create so much irritation, accompanied by fits of suffocation, that it was thought most prudent to desist for the present.

He thus continued, without apparently getting either better or worse, for two days longer: the only thing which he complained of was the excessive thirst, which he still attempted to allay by taking water into the mouth, which instantly flowed through the wound. In this way he consumed several gallons of water daily, and, if left for a moment without a supply of it, he became greatly excited and distressed. On asking him if he suffered from want of food, he wrote on the board that he did not, and that he thought he could do without any for several days longer.

Upon the fifth day after his admission, an attempt was again made to pass a tube into the gullet, which, after a few trials, was attended with success, and a small quantity of beef-tea was thrown into the stomach: it produced a slight degree of nausea, and the tube was withdrawn. In the evening the tube was again passed, and a little more broth was injected. He explained by writing that he did not feel any better for the nourishment he had received, but, on the contrary, rather exhausted. He complained of cold, and requested to have some more bedding, and was very anxious to be allowed to get up, and sit by the fire.

During the night he had a little sleep, and appeared more comfortable than usual; but at an early hour the following morning the nurse found him dying, and before I could be called he had expired.

The body was examined on the following day. Upon examining the wound in the throat, it was found that the main trunks of all the arteries of the neck had escaped injury, but the incision was within a line of the lingual artery and nerve on both sides. The epiglottis was divided transversely, and a portion of it remained attached to the root of the tongue; the pharynx had fallen down or retracted, when divided, so as to be below the incision. The viscera of the thorax were perfectly healthy; in the abdomen, the small intestines were found quite empty, and seemed shrunk: in the lower part of the colon and rectum, a small portion of hardened fæces remained; in the right lobe of the liver two immense cavities were found filled with fluid, apparently scrofulous matter mixed with bile, and several large hydatids. The largest of these abscesses was situated towards the convex surface of the liver, and was pointing through the diaphragm: it appeared to have been formed by the union of two smaller ones, as there was the remains of a septum in the centre; the walls were thick, and had a cartilaginous feel. The smaller abscess contained several hydatids of a much larger size than those in the other abscess; there was also a greater proportion of bile. It is singular that disease to such an enormous extent should have existed so long as this necessarily must have done, without it being ever suspected that he had any internal complaint, as his friends assured me was the case. The larger abscess would, no doubt, in a very short period have emptied itself in the cavity of the chest, and he would most probably have died from empyema.

There are two physiological facts which the above case serves well to illustrate: first, that the glottis possesses a power of guarding the passage into the trachea, independent of the epiglottis; for in this case the epiglottis was cut completely across, so as to be quite detached from the larynx, and in his attempts to drink the water all passed over the top of the larynx, without a drop entering or creating any considerable irritation; and, secondly, that the sensation of thirst is only *sympathetically* referred to the throat, but is, in reality, as much a sensation of the stomach as hunger; for, as has been said above, he passed quantities of water through the throat out at the wound, but could not allay the thirst, as none would pass into the stomach.

